# Psychometric properties of the Korean version of the Health Literacy on Social Determinants of Health Questionnaire (K-HL-SDHQ)

**DOI:** 10.1371/journal.pone.0224557

**Published:** 2019-11-18

**Authors:** Mikyeong Cho, Hyeonkyeong Lee, Young-Me Lee, Ja-yin Lee, Haeyoung Min, Youlim Kim, Sookyung Kim

**Affiliations:** 1 Mo-Im Kim Nursing Research institute, College of Nursing, Yonsei University, Seoul, South Korea; 2 School of Nursing, DePaul University, Chicago, Illinois, United States of America; 3 College of Nursing, Gyeongsang National University, Jinju, South Korea; University of Lleida, SPAIN

## Abstract

The association between the social determinant of health (SDH) and sustainable development goals, has directed attention toward the influence of SDH. However, there is a lack of evidence regarding the instruments used to assess SDH. Thus, this study was conducted to assess the validity and reliability of the Korean Version of the Health Literacy on Social Determinants of Health Questionnaire (K-HL-SDHQ). A total of 660 workers in Korea participated in an online survey. The K-HL-SDHQ measures four dimensions (Access, Understand, Appraise, and Apply) with 33 items. The HL-SDHQ was translated into Korean using the forward-back translation method. To test the validity and reliability of the Korean translated HL-SDHQ, item analysis for the 33 items was conducted. Internal consistency was examined using Cronbach’s α, an exploratory factor analysis, and a confirmatory factor analysis. The scale-level content validity index (S-CVI)/universal agreement of this study was .12 and S-CVI/average was .83 (item-CVI range = .50–1.00). The goodness of fit determined through a confirmatory factor analysis of the four dimensions was acceptable (χ^2^ (489) = 1475.054, *p* < .001, RMSEA = .06, CFI = .87, TLI = .85). The K-HL-SDHQ also demonstrated satisfactory internal consistency reliability (Cronbach’s α = .92). The findings indicate that the K-HL-SDHQ is a valid and reliable tool that can be used to assess the SDH of workers in Korea. It is suggested that this tool can be applied through repeated research with workers and non-workers for health promotion, and to enhance researchers’ understanding of the different levels of the HL-SDHQ.

## Introduction

Globally, health inequities due to the influence of social determinants of health (SDH) are a crucial issue warranting urgent actions to provide interventions for healthy living and wellbeing for all. SDH have gained recognition since the proposition of sustainable development goals (SDG) in 2015. The G20 Action Plan [1] and the World Health Organization’s (WHO) Shanghai declaration on promoting health in 2017 [2] acknowledged SDH as a necessary component for SDG by 2030. SDH are defined as a “condition in which people are born, grow, work, live, age and the wider set of forces and systems shaping the conditions of daily life,” and are primarily responsible for health inequities according to the WHO [3]. They are considered as essential determinants that influence individuals’ health behaviors and health status. The SDH approach includes physical and social environments from multiple sectors to confront health inequalities, such as occupational and environmental hazards, housing, water and sanitation systems, air quality, and climate change [4]. The effects of SDH have been researched over time by previous studies [5,6], and vary depending on the social status level including education, ethnicity, gender, income, occupation, and sexual orientation. Moreover, evidence shows that SDH account for 45–60% of the variation in health status [7], signifying the importance of SDH in improving health equity. However, access to health care should not differ according to personal characteristics such as gender, ethnicity, or socioeconomic status [8]. Therefore, we are certain that socially disadvantaged subgroups need more intensive services to improve health outcomes.

According to WHO’s Commission on SDH, health literacy is considered a major determinant of health status for all individuals, and they present a conceptual framework for SDH [6]. Health literacy refers to the ability to properly acquire, process, and understand basic health information and health services that individual needs to make appropriate decisions regarding health care [9]. There is growing recognition that health literacy is related to health behaviors and outcomes, and health-literacy-focused research, such as e-health information literacy and mental health literacy, have been widely investigated [10,11]. For example, people with low health literacy have difficulty in understanding the information related to health, experience difficulties in maintaining health, and fail to make timely decisions affecting health outcomes [12].

Health disparity depends on social, economic, and political factors, such as differences in lifestyle, environment, and medical care environment. Therefore, in order to reduce health disparities, it is necessary to acquire health information regarding SDH [13]. That is, people need to be able to access, understand, appraise, and apply information on SDH. Health literacy on SDH (HL-SDH) refers to a combination of access to health information (health promotion) and the ability to understand, appraise, and apply such information [14]. According to the American Hospital Association, HL-SDH is considered an important factor determining the health level and comprehensive care of individuals, and includes information on a series of resources such as food, housing, and transport provided to people in a community. Although policy advocacy and raising public awareness about SDH are important [6], there is little public awareness about the influence of SDH. Specifically, it is essential to invest in training workers for HL-SDH because workers’ health is influenced by and can influence the effect of other social determinants such as employment conditions, social status, financial security, and protection from physical and psychosocial hazards [5].

In Korea, there is no research focusing on HL-SDH. In order to improve the literacy level regarding Korean SDH, appropriate measurement tools are needed to judge the level of health literacy of Koreans. This suggests that it is important to use a valid tool for assessing the level of the HL-SDH. Therefore, this study aims to 1) translate the HL-SDH Questionnaire (HL-SDHQ) developed by Matsumoto and Nakayama [13] to Korean for use with the adult Korean working population and 2) to evaluate the validity and reliability of the translated Version of the HL-SDHQ.

## Materials and methods

### Study design

This descriptive study was designed to ensure that the translation of the HL-SDHQ [13] into Korean was methodologically sound for use for the Korean population. The HL-SDHQ was originally developed by Matsumoto and Nakayama [13], two Japanese scholars who measured health literacy regarding SDH among workers. The original tool was found to be valid and reliable.

### Participants

A convenience sample of 660 volunteers who registered to participate in an online panel in Korea was selected. Because most of the participants in the original study were workers, we also limited the sample to adults aged 20–64 years who were currently employed. Using a nonproportional quota sampling method, the sample was selected across regions (metropolitan areas and non-metropolitan), age groups, and gender. A previous study [15] suggested that the instrument related to health literacy does not appropriately identify the SDH of the elderly population in terms of their ability to understand and apply knowledge. Therefore, this present study excluded people over the age of 65 years. For the factor analysis, the sample size met the recommendation with a ratio of at least 20 cases per item [16].

### Instrument

The HL-SDHQ was developed in Japan in 2017 by Matsumoto and Nakayama, and its validity and reliability with Japanese workers were examined [13]. The HL-SDHQ includes 33 items in 4 dimensions: Access (n = 7), Understand (n = 7), Appraise (n = 7), and Apply (n = 12). Each item is rated on a 4-point Likert scale ranging from “1 = very difficult” to “4 = very easy.” In addition, to help assess participants’ level of comprehension, an additional response category of “don’t know/not applicable” was included and treated as a missing value. It was imputed by the average of each item following the guideline of the initial instrument developers. Each participant’s score was summed and divided by the total number of items. Higher scores indicated higher HL-SDH. In addition, demographic and socioeconomic characteristics of participants including gender, age, living area, size of residential area, education level, marital status, living alone, and religion were also assessed.

### Translation procedure

The HL-SDHQ, was originally written in Japanese, and did not examine the psychometric property tests of the English version of HL-SDHQ at the time of this study. Thus we were advised to translate the Japanese version to the Korean version by the original instrument developer. The Committee approach translation method is recommended to be appropriate in cases where linguistic and cultural differences are distinct [17]. In this study, however, the forward-back translation technique [18] was used because it was not available to have committee members who are have Japanese and Korean language expertise, in-depth knowledge of the social determinant health field, and translation process.

In the initial step, a bilingual translator, fluent in both Korean and Japanese and had an understanding of the cultural differences the two countries translated the Japanese version into Korean and then, another Korean-Japanese bilingual translator who did not know the original version, translated the Korean version back into Japanese. The exact meaning of the words and phrases are partly determined by cultural factors, so we instructed the translators to consider the different cultural and institutional contexts between the two countries. Next, an authorized Japanese translator compared the back translation with the original version, reviewed the differences and discrepancy in content, and confirmed the linguistic and conceptual equivalence of the Korean and Japanese versions. The Korean version was finally reviewed and reconfirmed for content clarity and cultural relevance by the first and second authors based on the English version reported by the instrument developer. Finally, a doctoral student majoring in Korean linguistics, reviewed the Korean version for the norms of the language and confirmed a final version. This version was named the Korean Health Literacy-Social Determinants of Health Questionnaire (K-HL-SDHQ).

The final version of the K-HL-SDHQ was tested with ten workers of different ages to ensure that the items were easily understandable, appropriate, and culturally sensitive. The Korean version questionnaire was emailed, and feedback was received by email or in person. One individual stated that the response categories ranging from “very easy” to “very difficult” were not familiar. However, most participants completed the questionnaire in 10–15 minutes without any assistance.

### Data collection

The study was approved by the Institutional Review Board of the affiliated institution prior to initiating the study (IRB No. Y-2018-0001). Data were collected from voluntarily registered participants through an internet research panel institute, from May 30 to June 7, 2018. Before starting the online survey, the purpose of the research, anonymity and confidentiality were explained on the first screen. It was designed to begin the survey by clicking the “Next” button, indicating agreeing to participate voluntarily in the survey. When the questionnaire was completed, participants were given a token of appreciation for responding to the online survey.

### Statistical analysis

The data were analyzed using SPSS 23 and AMOS 23. The participants’ demographic characteristics were expressed as numbers, means, standard deviations (SD), and percentages. For item analysis, the mean, standard deviation, and item-total correlation (ITC) were used. The content validity of the K-HL-SDHQ was tested by calculating an item-level content validity index (I-CVIs) and a scale-level content validity index (S-CVI) according to Polit and Beck’s recommendation [19]. In order to measure the content validity of the K-HL-SDHQ items, we invited 10 nursing professors who have teaching or research experience pertaining to SDH. Each item was evaluated using a 4-point Likert scale (1 = highly irrelevant, 2 = slightly irrelevant, 3 = relevant, 4 = highly relevant) to calculate the CVI and K* (modified kappa). The K* was calculated using the formula suggested by Polit et al. [20] to estimate the I-CVIs. Finally, S-CVI/universal agreement (UA) and S-CVI/average (Ave) were calculated to derive the S-CVI. The criteria used for evaluating each index are as follows: I-CVI ≥ .78, S-CVI/UA and S-CVI/Ave ≥ .80 [19].

To assess construct validity, an exploratory factor analysis (EFA) and confirmatory factor analysis (CFA) were conducted. EFA was performed to support construct validity by examining the characteristics of the factors and concept correspondence of each item [21]. EFA was conducted after following the Kaiser-Mayer-Olkin (KMO) measure and Bartlett’s test of sphericity using the principal component method from Oblimin, an oblique rotation. After that, CFA was performed to assess model fit, convergent validity, and discriminant validity. CFA involved root mean square error of approximation (RMSEA), comparative fit index (CFI), Tucker-Lewis Index (TLI), and chi-squared test. The RMSEA is a measure of the average residual variance and covariance; good models have RMSEA values that are equal to or less than .08. The CFI is an index that falls between 0 and 1, with values greater than .90 considered to be indicators of good fitting models. Convergent validity indicates if the latent variables are consistently measured well. To present convergent validity, a model should have a factor loading value (λ) ≥ 0.5, significance *p* < .05, average variance extracted (AVE) ≥ 0.5, and construct reliability (CR) ≥ 0.7 [22]. Discriminant validity was assessed when the AVE was more than the squared correlation coefficients [23]. To test the reliability of the Korean translated version, internal consistency using Cronbach’s α for the items in each factor and for all items as a whole was assessed.

## Results

### General characteristics of the sample

The mean age of the participants was 44.20 years with a standard deviation of 13.08 years. About 82.0% were college graduates, 56.5% were married, 20.5% were living alone, and 50.5% had no religion. Regarding the type of occupation, 39.1% of the participants were engaged in office work, 14.5% in service, and 13.9% in manual labor (Table 1).

**Table 1:** General characteristics of the participants (n = 660).

Characteristics	Categories	n (%) or M±SD
Age in categories (year)	20–29	132 (20.0)
	30–39	132 (20.0)
	40–49	132 (20.0)
	50–59	132 (20.0)
	60–64	132 (20.0)
Age (mean ± SD) (year)		44.20±13.08
Gender	Male	330 (50.0)
	Female	330 (50.0)
Living area	Metropolitan	330 (50.0)
	Non-metropolitan	330 (50.0)
Size of residence area	Large sized city	429 (65.0)
	Small and medium-sized cities	202 (30.6)
	Small sized city	29 (4.4)
Education level	≤High school	119 (18.0)
	≥College	541 (82.0)
Marital status	Married	373 (56.5)
	Single	225 (34.1)
	Divorces or separated	62 (9.4)
Living alone	Yes	135 (20.5)
	No	525 (79.5)
Religion	Yes	327 (49.5)
	No	333 (50.5)
Occupation	Office worker	258 (39.1)
	Service/sales	96 (14.5)
	Blue collar	92 (13.9)
	Teacher	52 (7.9)
	Professional job	50 (7.6)
	Freelance	45 (6.8)
	Administrative	42 (6.4)
	Public servant	25 (3.8)

### Item analysis

An analysis of the 33 items of the K-HL-SDHQ showed that the mean of the total item scores was 1.92–3.05. Item 12: “Understand that work that is not stable becomes a huge stress” scored the highest. Item 27: “Approach the manager or the employer regarding rewards that do not match efforts done at work” scored the lowest. ITC values ranged from .316 to .570, which is considered acceptable. As a result, all 33 items were used in this study [24] (Table 2).

**Table 2:** Items analysis of the Korean version of the health literacy on social determinants of health questionnaire (HL-SDHQ) scale (n = 660).

Item		M±SD	Corrected item-total correlation
1	Find out about the impact of social position on health	2.38±0.76	.390
2	Find information related to the impact of the daily life of a mother on the growth of the child to be born	2.70±0.82	.472
3	Find someone who is isolated from society and whose health is failing	2.18±0.81	.374
4	Find information on the relation between unemployment and stress	2.59±0.82	.452
5	Find out the support required by someone in trouble in the community or workplace	2.32±0.78	.433
6	Find out smoking is not going to eliminate the cause of stress	2.70±0.85	.514
7	Find information about the relationship between dietary changes and health	2.87±0.81	.562
8	Understand that the lesser the income the greater the tendency to become ill	2.64±0.83	.457
9	Understand that abuse suffered as a child has an impact even when one becomes an adult	2.99±0.85	.539
10	Understand that being isolated from the community and workplace impacts health	2.78±0.82	.570
11	Understand that determining how to proceed working on one’s own is related to stress	2.66±0.81	.569
12	Understand that work that is not stable becomes a huge stress	3.05±0.86	.514
13	Understand that widening income disparities dilute the ties between people	2.91±0.84	.499
14	Understand that in a society with a high level of stress, there is a tendency toward dependency on drugs	2.79±0.86	.521
15	Judge what inequities exist in society in view of living a healthy life	2.55±0.82	.474
16	Judge what kind of government services should be supplied to those really in need of support	2.42±0.81	.536
17	Judge what level of burden of work has on health	2.51±0.87	.521
18	Judge what kind of support should be supplied to someone in trouble in the community or workplace	2.36±0.75	.526
19	Judge how neighbors should help each other	2.47±0.79	.505
20	Judge the merits and demerits of the spread of processed foods	2.48±0.82	.511
21	Judge the kind of impact that motorization has on health	2.56±0.82	.449
22	Cooperate in the creation of a fair society in which everyone can live a healthy life	2.33±0.84	.458
23	Involve oneself in politics and public administration to help small children live a healthy life	2.23±0.80	.496
24	Participate in childcare support activities	2.32±0.78	.428
25	Participate in activities to eliminate poverty	2.21±0.82	.484
26	Involve oneself in politics and public administration to protect the health of workers both institutionally and legally	2.06±0.83	.486
27	Approach the manager or the employer regarding rewards that do not match efforts done at work	1.92±0.79	.316
28	Participate in activities to increase employment and vocational training opportunities	2.26±0.75	.464
29	Participate in activities that support an individual, including his or her family, who is in trouble in the community or workplace	2.23±0.76	.501
30	Participate in activities to spread the importance of ties with people for health	2.40±0.76	.453
31	Involve oneself in politics and public administration to make it easier for persons who have used illegal drugs to receive treatment	2.07±0.78	.443
32	Participate in activities that promote a healthy diet	2.64±0.79	.476
33	Involve oneself in politics and public administration to seek road priority for pedestrians and cyclists	2.29±0.81	.465

### Content validity

Ten experts evaluated content validity for the 33 items. I-CVI values ranged from 0.50 to 1.00, and 13 items that occupied the largest proportion of total items had an I-CVI of .90, followed by .80 for 8 items, and .70 for 7 items. Twenty-five items had I-CVI values ≥ .78 and K* >.74, which indicated excellent validity as interpreted by Halek et al. [25]. Seven items had I-CVI values < .78 and ≥ .60 and K* ≤ .74, which was regarded as indicating good validity. Only one item was found to have I-CVI values < .60 and ≥.40 and K* ≤ .59, indicating fair validity.

I-CVI for 7 items was .70 was .50 for 1 item in this study. The values satisfied the standards which suggested that I-CVI values less .50 are unacceptable [20]. Therefore, all the 33 items were used in this study. The S-CVI/Ave was .83, making the content validity acceptable [19] (Table 3).

**Table 3:** Content validity by ten experts (n = 10).

Item	Relevance of the questions				
	Number of ratings of 3 or 4	I-CVI^a^	P_c_^b^	K*^c^	Evaluation^d^
1	9	0.90	0.010	0.90	****
2	9	0.90	0.010	0.90	****
3	7	0.70	0.117	0.66	***
4	10	1.00	0.001	1.00	****
5	7	0.70	0.117	0.66	***
6	7	0.70	0.117	0.66	***
7	8	0.80	0.044	0.79	****
8	10	1.00	0.001	1.00	****
9	8	0.80	0.044	0.79	****
10	10	1.00	0.001	1.00	****
11	7	0.70	0.117	0.66	***
12	10	1.00	0.001	1.00	****
13	9	0.90	0.010	0.90	****
14	9	0.90	0.010	0.90	****
15	9	0.90	0.010	0.90	****
16	7	0.70	0.117	0.66	***
17	9	0.90	0.010	0.90	****
18	9	0.90	0.010	0.90	****
19	8	0.80	0.044	0.79	****
20	9	0.90	0.010	0.90	****
21	9	0.90	0.010	0.90	****
22	9	0.90	0.010	0.90	****
23	8	0.80	0.044	0.79	****
24	7	0.70	0.117	0.66	***
25	8	0.80	0.044	0.79	****
26	9	0.90	0.010	0.90	****
27	5	0.50	0.246	0.34	**
28	8	0.80	0.044	0.79	****
29	8	0.80	0.044	0.79	****
30	9	0.90	0.010	0.90	****
31	7	0.70	0.117	0.66	***
32	8	0.80	0.044	0.79	****
33	9	0.90	0.010	0.90	****
S-CVI/Ave^e^ 0.83					
S-CVI/UA^f^ 0.12					

^a^I-CVI (Item-level content validity index) = number of experts providing a rating of 3 or 4/number of experts

^b^P_c_ (Probability of chance occurrence) = [N!A!(N-A)!] * 0.5^N^, N = number of experts; A = number of experts agreeing on a rating of 3 or 4

^c^K* (Modified kappa) = (I-CVI- P_c_)(1- P_c_)

^d^Evaluation criteria for the level of content validity; relationship between I-CVI and K*; excellent validity = I-CVI≥0.78 and K*>0.74(****); good validity I-CVI < 0.78 and ≥0.60 and K*≤0.74 (***); fair validity I-CVI < 0.6 and ≥0.40 and K*≤0.59(**); and poor validity I-CVI < 0.4 and K*<0.40(*)

^e^S-CVI/Ave = Scale-level content validity index/average

^f^S-CVI/UA = Scale-level content validity index/universal agreement.

### Construct validity

Regarding the EFA of the 33 items, the KMO was high at .93 [26]. Bartlett’s test of sphericity showed statistically significant results for EFA (KMO = .93, χ^2^ = 7703.79, *p* < .001). The EFA was conducted using a principal component method to minimize data loss during factor extraction. Oblique rotation was performed by considering characteristics of HL-SDHQ that could be correlated with each item. Four fixed factors were extracted each with eigenvalues > 1.00. Items considered for these four factors were those with a loading value greater than 0.2 [27]. Factor details are shown in Table 4.

**Table 4:** Result of exploratory and confirmatory factor analysis: Social determinants of the health literacy (n = 660).

Domain	Item	EFA				CFA		
		Factor 1	Factor2	Factor 3	Factor4	Factor Loading	AVE	CR
Under stand	1. Understand that the lesser the income the greater the tendency to become ill (8)	.553				.545	.55	.90
	2. Understand that abuse suffered as a child has an impact even when one becomes an adult (9)	.762				.764		
	3. Understand that being isolated from the community and workplace impacts health (10)	.734				.742		
	4. Understand that determining how to proceed working on one’s own is related to stress (11)	.647				.657		
	5. Understand that work that is not stable becomes a huge stress (12)	.780				.726		
	6. Understand that widening income disparities dilute the ties between people (13)	.753				.682		
	7. Understand that in a society with a high level of stress, there is a tendency toward dependency on drugs (14)	.672				.627		
Apply	8. Cooperate in the creation of a fair society in which everyone can live a healthy life (22)		.388			.478	.46	.91
	9. Involve oneself in politics and public administration to help small children live a healthy life (23)		.546			.579		
	10. Participate in childcare support activities (24)		.600			.553		
	11. Participate in activities to eliminate poverty (25)		.646			.620		
	12. Involve oneself in politics and public administration to protect the health of workers both institutionally and legally (26)		.715			.685		
	13. Approach the manager or the employer regarding rewards that do not match efforts done at work (27)		.595			.539		
	14. Participate in activities to increase employment and vocational training opportunities (28)		.700			.637		
	15. Participate in activities that support an individual, including his or her family, who is in trouble in the community or workplace (29)		.715			.665		
	16. Participate in activities to spread the importance of ties with people for health (30)		.680			.602		
	17. Involve oneself in politics and public administration to make it easier for persons who have used illegal drugs to receive treatment (31)		.665			.626		
	18. Participate in activities that promote a healthy diet (32)		.542			.487		
	19. Involve oneself in politics and public administration to seek road priority for pedestrians and cyclists (33)		.677			.619		
Appraise	20. Judge what inequities exist in society in view of living a healthy life (15)			.471		.527	.43	.84
	21. Judge what kind of government services should be supplied to those really in need of support (16)			.620		.599		
	22. Judge what level of burden of work has on health (17)			.619		.605		
	23. Judge what kind of support should be supplied to someone in trouble in the community or workplace (18)			.623		.608		
	24. Judge how neighbors should help each other (19)			.559		.572		
	25. Judge the merits and demerits of the spread of processed foods (20)			.256		.561		
	26. Judge the kind of impact that motorization has on health (21)			.445		.540		
Access	27. Find out about the impact of social position on health (1)				.237		.41	.82
	28. Find information related to the impact of the daily life of a mother on the growth of the child to be born (2)				.494			
	29. Find someone who is isolated from society and whose health is failing (3)				.575			
	30. Find information on the relation between unemployment and stress (4)				.723			
	31. Find out the support required by someone in trouble in the community or workplace (5)				.730			
	32. Find out smoking is not going to eliminate the cause of stress (6)				.504			
	33. Find information about the relationship between dietary changes and health (7)				.590			
	Eigen value	9.16	3.31	1.51	1.32			
	Explained variance(%)	27.76	10.03	4.58	3.99			
	Cumulative (%)	27.76	37.80	42.37	46.36			
Kaiser-Meyer-Olkin (KMO) = .93; Bartlett’s test of sphericity = 7703.79 (*p* < .001)						Model fitness χ^2^ (489) = 1475.054, *p* < .001, RMSEA = .06, CFI = .87, TLI = .85		
	Cronbach’s α	.86	.86	.77	.75			
Total Cronbach’s α = .92								

EFA = Exploratory factor analysis; CFA = Confirmatory factor analysis; AVE = Average variance extracted; CR = Composite reliability; RMSEA = Root mean square error of approximation; CFI = Comparative fit index; TLI = Tucker-Lewis index

The factor loading was .553-.780 for factor 1, .388-.715 for factor 2, .256-.623 for factor 3, and .237-.730 for factor 4, which satisfied the standard [27]. The factor loadings were consistent with the corresponding domains in the original version. The explanatory power was 46.36% of the total variance, 27.76% for factor 1, 10.03% for factor 2, 4.58% for factor 3, and 3.99% for factor 4. These extracted factors were verified and named similar to that of the original version (Understand, Apply, Appraise, and Access) (Table 4). Based on the EFA results, 4 components were extracted. Then, CFA was conducted using maximum likelihood to examine model fit. The findings showed that the model fit was good: χ^2^ (489) = 1475.054, *p* < .001, CFI = .87, TLI = .85, RMSEA = .06 [22] (Table 4).

To test construct validity, convergent validity and discriminant validity were examined, which showed a low correlation and independence between sub-factors. First, convergent validity was .412-.764. When analyzed using AVE, the convergent validity was between .41-.55, and CR was between .82-.91, which partially met the standard of convergent validity. Second, discriminant validity was assessed which showed low correlation and independence between sub-factors. AVE was smaller than the squared correlation between Factors 1 and 2 and Factors 1 and 3, and so the criterion for acceptable discriminant validity was partially satisfied [23].

### Reliability

Cronbach’s α, which was .92 in Matsumoto and Nakayama’s HL-SDHQ [13], was confirmed as .92 in the present study as well. Four subdomains of Understand, Apply, Appraise, and Access showed Cronbach’s α values of .86, .86, .77, and .75, respectively (Table 4). Considering the large difference in the education level of the participants, the internal validity was examined by the education level. The findings showed minuscule differences; Cronbach’s α was .92 in those with college education and .91 in those with less education, indicating that the instrument is equally appropriate for both groups.

## Discussion

HL-SDHQ was developed originally in Japanese as a tool to measure and access health information regarding social determinants of health as well as to understand, evaluate, and apply this health information to promote health [13]. This study is the first translation after the development of the original instrument; there is no other language version yet. After the translation, the validity and reliability of the Korean version of HL-SDHQ was tested with an appropriate sample size of 660 Korean workers in Korea. The ITC between the items of analysis and total score was over .20; thus, 33 items were accepted and used in this study [13]. The 4 factors had the same theoretical structure in the EFA as they did in the original instrument. By linking SDG to health literacy, greater attention is now being paid to the influence of SDH on population health. However, there is still very little awareness of the ability of SDH. Therefore, it would be worthwhile to first introduce K-HL-SDHQ to SDH researchers and provide them with evidence for the applicability of HL-SDHQ to other countries.

The 7 items from factor 1, Access category, in the original tool were extracted as factor 4 in this study, and the final 7 items were composed. Items 27, 28, 32, and 33 (original items—1, 2, 6, and 7) overlap with the Understand category in the original factor 1. This might be because “know”, “find”, and “understand” were considered as the same concept. The 7 items in factor 2, Understand category, of the original tool were extracted as factor 1 comprising 7 items. Factor 3 consisted of 7 items, and the 7 items in the Appraise category were extracted from the 3 factors of the study tool to comprise the final 7 items. It is assumed that items 15 and 20 included in the Understand category of Factor 1 are also in this study; it is considered bound to both sides because it is assumed to be a premise to understand the judgment. The 12 items in the Apply category of factor 4 in the study tool were extracted as factor 2 and the final 12 items were composed. “22. Cooperate in the creation of a fair society in which to live a healthy life” was included in the third factor Appraise on the assumption that cooperation is possible through the appraise process.

CFA confirmed the original 4-factor structure of HL-SDHQ, indicating satisfactory construct validity [28]. The criteria of convergent validity include ≥ .70 CR and ≥ .50 AVE. In this study, CR values were ≥.70 for all factors, indicating adequate reliability of internal consistency. Except for 2 factors, the AVE extracted was ≥.50, which means that the amount of variance is a too small to explain the latent construct. However, Fornell and Larcker [22] suggested that the use of CR alone is enough for confirming convergent validity. Thus, this study confirmed convergent validity of K-HL-SDHQ. In this study, the structure extracted was the same as the original tool; however, in the content validity test, the CVI of the 27th item (“Approach the manager or the employer regarding rewards that do not match efforts done at work”) was found to be lowered to .50. This may be due to differences in the cultural backgrounds of the experts involved, which may affect the factor structure [29]. In Korean society, it is difficult to question the unreasonableness in the workplace as workers are reluctant to go directly to their employers even if they experience difficulties. Therefore, it is reasonable to consider this as one of the causes of low content validity. However, continued exposure to these organizational cultures increases job stress and is a major contributor to health risk factors such as smoking, drinking, and insomnia [30]. Therefore, as part of SDH, understanding the influence of the aforementioned item on health is an important approach [13,14]. Thus, it may be reasonable to retain the item for the worker population, which in turn suggests further research on the non-worker population.

In evaluating the internal consistency of the instrument, Cronbach's α for all the items was .92, which indicated it to be a highly reliable instrument. Cronbach’s α ranged between .75-.86 in the reliability analysis of the four subscales. Therefore, it has proven to be a suitable tool with acceptable internal consistency reliability to measure the level of literacy regarding the determinants of Korean social health. In addition, the K-HL-SDHQ is a valid and reliable tool extracted from 4 factors similar to the original tool at the time of development. The validity and reliability of the K-HL-SDHQ were confirmed in this study. Thus, the results of this study are helpful to understand the level of HL and its social determinants among Korean workers and enable the comparison of the findings with the social HL levels of workers in other countries. Furthermore, the findings of this study can be used as basic data for developing strategies to improve health promotion behavior; more specifically, by focusing on the literacy level among decision makers in the field of social health.

This study has certain limitations. First, the participants were not diverse, as the sample was confined to workers aged between 20 and 64 years. EFA was used to test construct validity but extracted factors by eigenvalue rather than through a theoretical basis. The results are likely to vary according to the characteristics of the collected sample [31]. Therefore, it is necessary to confirm the validity of the tool according to the changes in the target group. It is also necessary to reflect on cultural considerations and the possibility that bias may have been involved in the process of evaluating the validity of the translated tools [32]. In this study, only nursing professors who had experience in research and education of SDH were examined. Thus, it is recommended that future research seek to examine content validity by consulting various experts with different backgrounds.

## Conclusions

This study attempted to translate the HL-SDHQ [13] into Korean, which was originally developed in Japanese in 2017, and to test its validity and reliability so that it can be used to assess the literacy levels of workers' SDH. Results of the reliability and validity test of the K-HL-SDHQ revealed that the tool was valid and suitable for measuring HL-SDH among Korean workers. Based on the results in this study, it is possible to conduct comparative studies with other countries with different cultural characteristics. It is also expected that the tool can be used in the screening of literacy levels of Korean SDH and in developing interventions to improve the literacy of Korean SDH. It is suggested that this tool be applied through repeated research on workers and non-workers, so that the applicability and usefulness of the tool can be expanded.

## Supporting information

S1 Appendix Korean version of the health literacy on social determinants of health questionnaire. (PDF)
