# Supplementary material for: Psychometric properties of the Korean version of the Health Literacy on Social Determinants of Health Questionnaire (K-HL-SDHQ)
Source: PLoS One. 2019 Nov 18;14(11):e0224557. doi: 10.1371/journal.pone.0224557 (PMC6860445; doi:10.1371/journal.pone.0224557)
Supplement: S1 Appendix — (PDF) [file pone.0224557.s001.pdf]

# 사회적 건강결정인자 문해력

귀하께 아래의 사항을 인지 및 수행하는 것이 쉬운지 어려운지 여쭈겠습니다. 각각 (매우 쉽다)부터 (매우 어렵다)까지의 선택사항 중에서 가장 적합한 사항에 체크를 해주십시오. (각 문항 당 하나씩 체크하기)

| 문항 | | 매우 쉽다 | 약간 쉽다 | 약간 어렵다 | 매우 어렵다 | 모른다/해당 안된다 |
|---|---|---|---|---|---|---|
| (1) | 사회적 지위가 건강에 영향을 미친다는 것에 대해서 알기 | 1 | 2 | 3 | 4 | 5 |
| (2) | 임산부의 생활이 태어나는 아이의 성장에 미치는 영향에 관한 정보를 찾기 | 1 | 2 | 3 | 4 | 5 |
| (3) | 사회에서 고립되어 건강을 해치고 있는 사람을 찾기 | 1 | 2 | 3 | 4 | 5 |
| (4) | 노동자들의 실업과 스트레스의 관계에 관한 정보를 찾기 | 1 | 2 | 3 | 4 | 5 |
| (5) | 지역이나 직장에서 어려움을 겪고 있는 사람에게 필요한 지원에 대해서 알기 | 1 | 2 | 3 | 4 | 5 |
| (6) | 흡연이 스트레스 원인을 해결해주지 못한다는 것에 대해서 알기 | 1 | 2 | 3 | 4 | 5 |
| (7) | 식생활의 변화와 건강의 관계에 관한 정보를 찾기 | 1 | 2 | 3 | 4 | 5 |
| (8) | 소득이 적은 사람일수록 병에 걸리기 쉽다는 것을 이해하기 | 1 | 2 | 3 | 4 | 5 |
| (9) | 어릴 적에 받았던 학대가 성인이 되어도 영향을 줄 수 있다는 것을 이해하기 | 1 | 2 | 3 | 4 | 5 |
| (10) | 지역이나 직장에서 고립 상태로 있는 것이 건강에 영향을 미친다는 것을 이해하기 | 1 | 2 | 3 | 4 | 5 |
| (11) | 업무의 진행 방식을 스스로 결정할 수 있는 것은 스트레스와 연관이 있다는 것을 이해하기 | 1 | 2 | 3 | 4 | 5 |
| (12) | 업무의 고용 불안정성은 큰 스트레스가 된다는 것을 이해하기 | 1 | 2 | 3 | 4 | 5 |
| (13) | 소득 격차의 확대가 사람들의 관계를 멀어지게 한다는 것을 이해하기 | 1 | 2 | 3 | 4 | 5 |
| (14) | 스트레스가 많은 사회에서 약물에 대한 의존도가 높다는 것을 이해하기 | 1 | 2 | 3 | 4 | 5 |
| (15) | 사회에서 건강한 생활을 하는데 있어서 어떠한 불공평함이 존재하는지 판단하기 | 1 | 2 | 3 | 4 | 5 |
| (16) | 도움이 정말로 필요한 사람에게 어떠한 행정적 서비스가 제공되어야 하는지 판단하기 | 1 | 2 | 3 | 4 | 5 |

| | | | | | | |
|---|---|---|---|---|---|---|
| (17) | 업무의 부담감이 어느 정도면 건강에 영향을 미치는지 판단하기 | 1 | 2 | 3 | 4 | 5 |
| (18) | 지역이나 직장에서 어려움을 겪고 있는 사람들에게 어떠한 지원을 제공해야 하는지 판단하기 | 1 | 2 | 3 | 4 | 5 |
| (19) | 이웃끼리 서로 어떻게 도움을 주면 좋을지 판단하기 | 1 | 2 | 3 | 4 | 5 |
| (20) | 가공식품 보급과 관련된 장단점을 판단하기 | 1 | 2 | 3 | 4 | 5 |
| (21) | 자가 운전 및 대중 교통 이용 증가가 건강에 어떠한 영향을 미치는지 판단하기 | 1 | 2 | 3 | 4 | 5 |
| (22) | 모두가 건강하게 지낼 수 있는 공평한 사회를 만들기 위해 협력하기 | 1 | 2 | 3 | 4 | 5 |
| (23) | 어린아이들이 건강하게 생활할 수 있도록 정치가나 행정 기관에 제도 개선을 요구하기 | 1 | 2 | 3 | 4 | 5 |
| (24) | 육아를 지원하는 활동에 참여하기 | 1 | 2 | 3 | 4 | 5 |
| (25) | 빈곤을 없애기 위한 활동에 참여하기 | 1 | 2 | 3 | 4 | 5 |
| (26) | 노동자들의 건강을 지키기 위한 제도 및 법률 개선, 제정을 정치가나 행정 기관에 요구하기 | 1 | 2 | 3 | 4 | 5 |
| (27) | 업무상의 노력과 적합하지 않은 보수와 관련해서 상사나 고용자들에게 요구하기 | 1 | 2 | 3 | 4 | 5 |
| (28) | 취직, 직업 훈련의 기회를 많이 늘리기 위한 활동에 참여하기 | 1 | 2 | 3 | 4 | 5 |
| (29) | 지역이나 직장에서 어려움을 겪고 있는 사람들이나 그 가족들을 도와주기 위한 활동에 참여하기 | 1 | 2 | 3 | 4 | 5 |
| (30) | 건강을 위해 사람과의 유대관계가 중요하다는 것을 널리 알리기 위한 활동에 참여하기 | 1 | 2 | 3 | 4 | 5 |
| (31) | 불법 약물을 사용한 사람들이 앞으로 치료를 더 받기 쉽게 할 수 있도록 정치가나 행정 기관에 요구하기 | 1 | 2 | 3 | 4 | 5 |
| (32) | 건강한 식생활을 위한 활동에 참여하기 | 1 | 2 | 3 | 4 | 5 |
| (33) | 보행자나 자전거 이용자들을 위한 전용 도로 확충을 정치가나 행정 기관에 요구하기 | 1 | 2 | 3 | 4 | 5 |
